# Supplementary material for: Identification of photoperiod-induced specific miRNAs in the adrenal glands of Sunite sheep (Ovis aries)
Source: Front Vet Sci. 2022 Jul 22;9:888207. doi: 10.3389/fvets.2022.888207 (PMC9354845; doi:10.3389/fvets.2022.888207)
Supplement: Supplementary file 1 [file Data_Sheet_1.ZIP › Supplementary file/miRDeep2 file.zip!/miRDeep2 file/Know_Structure/SPLP42A3/oar-mir-29b-2.pdf]

miRBase precursor : oar-mir-29b-2
Total read count : 4452
oar-miR-29b read count : 4061
remaining reads : 391

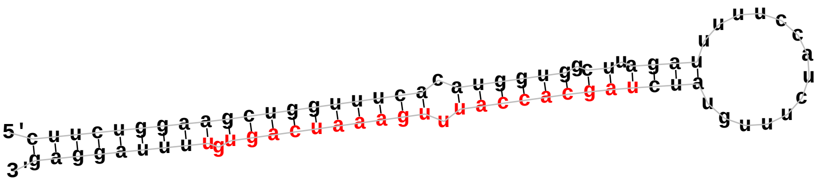

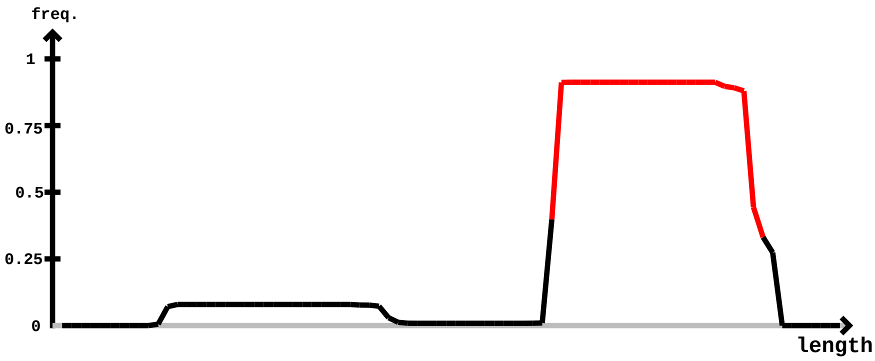

oar-miR-29b

```
5'- cuucuggaagcugguuucacaugguggcuuagauuuuuccaucuuuguaucuagcaccauuugaaaucaguguuuuaggag   -3'    exp
    (((((((((((((((((((.((((((.((.((((..............)))))))))))).)))))))))).)))))))))     reads      mm         sample
    .........gcugguuucacaugguggcuu...................................................     1          0          seq
    .........gcugguuucacaugguggcuua..................................................     1          0          seq
    .........gcugguuucacaugguggcuuag.................................................     1          0          seq
    .........gcugguuucacaugguggcuuaga................................................     20         0          seq
    ..........cugguuucacaugguggcuu...................................................     7          0          seq
    ..........cugguuucacaugguggcuua..................................................     1          0          seq
    ..........cugguuucacaugguggcuuag.................................................     16         0          seq
    ..........cugguuucacaugguggcuuaga................................................     192        0          seq
    ..........cugguuucacaugguggcuuagau...............................................     66         0          seq
    ..........cugguuucacaugguggcuuagauu..............................................     10         0          seq
    ..........cugguuucacaugguggcuuagauuu.............................................     1          0          seq
    ...........ugguuucacaugguggcuu...................................................     1          0          seq
    ...........ugguuucacaugguggcuuag.................................................     1          0          seq
    ...........ugguuucacaugguggcuuaga................................................     17         0          seq
    ...........ugguuucacaugguggcuuagau...............................................     12         0          seq
    ...........ugguuucacaugguggcuuagauu..............................................     5          0          seq
    .................................uuuuuccaucuuuguauA..............................     2          1          seq
    .................................uuCuuccaucuuuguauc..............................     1          1          seq
    .................................uuuuuccaucuuuguauU..............................     3          1          seq
    .................................uuuuuccaucuuuguauc..............................     29         0          seq
    .................................uuuuuccaucCuuguauc..............................     1          1          seq
    .................................uuuuuccaucuCuguauc..............................     1          1          seq
    ..................................uuuuccaucuuuguaucu.............................     1          0          seq
    ................................................aucuagcaccauuugaaaucagu..........     1          0          seq
    .................................................ucuagcaccauuugaaaucagu..........     4          0          seq
    ..................................................cuagcaccauuugaaauc.............     31         0          seq
    ..................................................cuaCcaccauuugaaauc.............     1          1          seq
    ..................................................cuagcaccauuugaaauca............     6          0          seq
    ..................................................cuagcaccauuugaaaucag...........     7          0          seq
    ..................................................cuagcaccauuugaaaucagu..........     1093       0          seq
    ..................................................cuagcaccauuugaaaucGgu..........     15         1          seq
    ..................................................cuagcaccauuugaaaucagug.........     387        0          seq
    ..................................................cuagcaccauuugaaaucagugu........     186        0          seq
    ..................................................cuagcaccauuugaaaucaguguu.......     3          0          seq
    ...................................................uagcaccauuugaaauU.............     2          1          seq
```

oar-miR-29b

```
cuucuggaagcugguuucacaugguggcuuagauuuuuccaucuuuguaucuagcaccauuugaaaucaguguuuuaggag
...................................................uagcaccauuugaaauc.............    33      0       seq
...................................................uagcaccauuugaaauA.............    2       1       seq
...................................................uagcaccauuugaaaucG............    10      1       seq
...................................................uagcaccauuugaaauca............    8       0       seq
...................................................uagcaccauuugaaaucag...........    13      0       seq
...................................................uagcaccauuugaaaucGg...........    32      1       seq
...................................................uagcaccauuugaaaucagu..........    586     0       seq
...................................................uagcaccauuugaaaucGgu..........    242     1       seq
...................................................uagcaccauuugaaaucagug.........    114     0       seq
...................................................uagcaccauuugaaaucagugu........    70      0       seq
...................................................uagcaccauuugaaaucaguguu.......    1212    0       seq
....................................................agcaccauuugaaaucaguguu.......    4       0       seq
```
